# Temporal changes in ruminal microbiota composition and diversity in dairy cows supplemented with a *lactobacilli*-based DFM

**DOI:** 10.3389/fvets.2025.1584959

**Published:** 2025-05-16

**Authors:** Bronwyn E. Campbell, Mohammad Mahmudul Hassan, Robert J. Moore, Timothy Olchowy, Martin Soust, Rafat Al Jassim, John I. Alawneh

**Affiliations:** ^1^School of Science, RMIT University, Bundoora, VIC, Australia; ^2^School of Veterinary Science, The University of Queensland, Gatton, QLD, Australia; ^3^Faculty of Veterinary Medicine, University of Calgary, Calgary, AB, Canada; ^4^Terragen Biotech Pty Ltd., Coolum Beach, QLD, Australia; ^5^Queensland Alliance for Agriculture and Food Innovation, Saint Lucia, QLD, Australia; ^6^Department of Primary Industries, Plant Biosecurity and Product Integrity, Biosecurity Queensland, Brisbane, QLD, Australia

**Keywords:** ruminal microbiota, dairy cows, direct-fed microbial, microbial diversity, temporal changes

## Abstract

**Introduction:**

This study aimed to evaluate the impact of lactobacilli-based direct-fed microbial (DFM) supplementation on the composition and diversity of the ruminal microbiota in dairy cows. Understanding how DFM influences microbial populations can inform strategies to enhance animal health and productivity.

**Methods:**

Over a 16-month period (September 2021 to January 2023), ruminal fluid samples were collected from fifty dairy cows assigned to either a DFM-supplemented group (DFM; *n* = 25) or an unsupplemented control group (CON; *n* = 25). Microbial DNA was extracted and subjected to 16S rRNA gene amplification and sequencing. Microbial diversity was assessed using alpha- and beta-diversity metrics (*p* < 0.05), and linear discriminant analysis effect size (LEfSe) was employed to identify differentially abundant taxa. Multivariable analyses were used to explore associations with age, average milk production, days in milk (DIM), time, and supplementation.

**Results:**

The dominant bacterial phyla identified were Bacillota and Bacteroidota, while Methanobacteriaceae was the predominant archaeal family. The DFM group showed significantly higher abundance of genera such as *Eubacterium_Q*, *Atopobium* sp. *UBA7741*, and *Sharpea* (*p* < 0.05). Conversely, *Bacillus_P_294101* and *SFMI01* were more abundant in the CON group. Temporal changes in microbial composition were observed, with significant differences in community diversity and structure between groups over time.

**Discussion:**

These findings demonstrate that lactobacilli-based DFM supplementation can significantly alter the ruminal microbial ecosystem in dairy cows. The observed microbial shifts, including increases in beneficial bacterial taxa, highlight the potential of DFM as a nutritional strategy to modulate rumen function and improve dairy cow performance.

## 1 Introduction

The rumen of dairy cows is a complex and vital ecosystem responsible for breaking down plant material and extracting essential nutrients for maintenance and production. This process is driven by a diverse microbial community consisting of bacteria, protozoa, phages, anaerobic fungi and archaea. These microbes ferment fiber, produce essential nutrients, and detoxify harmful substances (1, 2). Bacteria, particularly from Bacillota and Bacteroidota, dominate, while archaea and eukaryotes (protozoa and anaerobic fungi) form smaller portions of the community (3). Most of these microbes reside in the ruminal fluid, particle-associated rumen liquid, and solid phase of the digesta, with genera like *Succiniclasticum* and *Prevotella* playing key roles in fermentation (4). The ruminal microbiota offers valuable insights into digestive health and the microbial dynamics that influence digestion, complementing the understanding provided by the rumen itself (4). A common nutritional problem in ruminants is reduced feed efficiency due to imbalanced gut microbiota, leading to poor digestion and nutrient absorption. The experimental direct-fed microbial (DFM) formulation addresses this issue by introducing specific strains of probiotics that restore microbial balance, enhance enzymatic activity, and improve fiber breakdown. This targeted intervention promotes better nutrient utilization, improving feed efficiency and overall animal health (5, 6).

Recent research has highlighted the critical role of microbiota in livestock health, significantly influencing digestion, nutrient absorption, and overall production efficiency (3, 7). Zeineldin et al. (7) reported that cows with a wide variety of microbial species in the rumen experienced better overall health and productivity. Furthermore, maintaining microbial diversity through appropriate dietary management and DFM supplementation can enhance immune function and ultimately contribute to the sustainability of dairy farming practices. Ogunade et al. (8) examined the effects of different dietary interventions on ruminal microbiota composition but not on fermentation end-products or energy status of the experimental steers. The fiber-enriched diet serves as a substrate for microbial growth, and the DFM provides targeted microbial supplementation to maximize the fermentation and digestion process. Interactions between diet and age play a critical role in shaping the composition of the gut microbiota, driving changes in microbial populations (9, 10). Evidence suggests that dietary interventions can effectively modulate microbial communities, promoting improved health outcomes in cattle (11). Optimizing the diet can boost beneficial microbial populations, thereby enhancing digestive efficiency. Feed additives like DFMs are added to modulate the gut microbiota, aiming to enhance the health and productivity of dairy cows (12).

Lactobacilli-based DFM supplementation influenced microbial populations in the digestive tract to potentially enhance fermentation efficiency and nutrient absorption (13). Further investigation is warranted to better understand the functional implications of these changes and their long-term effects on the health and productivity of dairy cows. Despite its potential advantages in improving ruminant production and health, the impact of DFM supplementation on the rumen microbiota composition and diversity in dairy cows remains largely unexplored. Further research is needed to provide insights on ways to optimize DFM interventions and improve the health and productivity of dairy cows. This study aimed to analyze the microbial communities in the ruminal fluid samples (with digesta) of DFM-supplemented and un-supplemented (control) cows. Through a comparative analysis of these microbial communities over time, the research aims to quantify the temporal changes ruminal microbiota dynamics and identify specific taxa influenced by the DFM supplement. The hypothesis was that supplementation with the DFM would lead to beneficial shifts in the ruminal microbiota by changing the diversity and composition. This study provides insights on how dairy cows’ ruminal microbiota composition and diversity are altered over time when supplemented with a lactobacilli-based DFM.

## 2 Materials and methods

### 2.1 Study location and study herd

The complete details of the study location, herd, study design and production performances have been published by our team (14). This longitudinal, negatively controlled study, with a randomized design blocked on treatment groups, was conducted at a commercial dairy farm in Harrisville, Queensland, Australia, from September 2021 to January 2023. The milking herd, comprising approximately 350 Holstein cows, included the randomly selected study cows. The cow herd was managed in two groups for housing, feeding, and milking. Both groups followed a partial mixed ration (PMR) feeding system, where cows received a mixed ration on a covered feed pad during the day and grazed pasture at night. The composition of the mixed ration provided once daily consisted mainly of maize or barley silage, lucerne hay, soybean silage, canola meal, and barley or wheat grain (Supplementary Table S1). An additional 1.5 kg of grain was fed twice daily during milking. All cows in the study had free access to both water and their allocated pasture. Typical of Southeastern Queensland dairy production systems, the study cows were managed as two separate groups such that the pasture allowance (up to 6 kg of DM/cow per day) and the PMR feed were sufficient for the maintenance and production requirements of a 600-kg cow producing 35 L of milk/d. The target average dry matter intake (DMI) was 22 kg/cow/day. The pasture consisted of an 80:20 mix of ryegrass (*Lolium perenne*) and white clover (*Trifolium repens*). The chemical analysis of the total ration is reported by our team (14) and in Supplementary Table S1. The study farm had 11 well-defined grazing paddocks that were similarly managed concerning grazing time, rotation frequency, and the irrigation program. Each of these 11 paddocks was subdivided along its length to create 22 paired grazing subpaddocks, each approximately 1.5 ha in size. The paired subpaddocks were grazed for approximately 2 d, and then the cows moved to the next pair of subpaddocks according to the grazing rotation program. If required, the grazing period on any pair of subpaddocks was adjusted based on the consumption pattern of the cows. Both groups were housed in a single dry lot partitioned to provide separate feeding and loafing areas and free access to water. The DFM (Mylo®, Terragen Biotech, Queensland, Australia) group animals received an additional 10 mL/cow/day of a DFM supplement (manufacturer recommended dose), top-dressed onto their mixed ration using a 2 L manual pressure sprayer (245 kPa maximum pressure, Aqua Systems Australia). The DFM contained approximately 3.5 × 10^9^ CFU/mL each of three live bacterial strains: *Lentilactobacillus buchneri* Lb23, *Lactocaseibacillus casei* Lz26, and *Lactocaseibacillus paracasei* T9. The control group was not supplemented and only received a PMR diet.

### 2.2 Study animals

This study is part of a larger project and focuses on temporal dynamics and changes in the ruminal microbiota. The sub-group of the study herd was randomly selected as the study animals. Assuming a difference of 50% in microbiome structure, an alpha 2.5%, power of 80%, no change in rumen microbiome structure in the control animals, a change in the rumen microbiome structure in the DFM supplemented animals of 50%, and a difference of 15% is negligible, the minimum number of animals in each sub-group was 20. We further inflated the sample size by 25% to account for any loss of follow-up. A total of 50 Holstein cows (average body weight 590 ± 67 kg), including both primiparous and multiparous cows, were randomly selected based on parity and days in milk (DIM), and assigned into two sub-groups: control (*n* = 25) and DFM (*n* = 25). The study biodata was described by Ramirez-Garzon et al. (14) and provided in a Supplementary Table S2.

### 2.3 Sample collection

Cows were securely restrained in a chute with their heads manually restrained. An oral speculum, an oro-ruminal sampling tube, and a manually operated pump (Double action hand pump, Wanderer) were used to collect approximately 200 mL of ruminal fluid from each cow approximately 3 h post-diurnal feeding (9:00 a.m.). Samples were collected at approximately 2-month intervals at 8 points of time throughout the study, covering all stages of lactations. The fluid was collected into an Erlenmeyer vacuum filter flask. Ruminal fluid samples (with digesta) were placed into sterile 5 mL poly-propylene flat-bottom tubes (Interpath, Melbourne, Australia) and stored at −20°C before being transported to the laboratory on dry ice for analysis.

### 2.4 DNA extraction and PCR

DNA was extracted from ruminal samples using the Maxwell® RSC Fecal Microbiome DNA Kit (Promega, Fitchburg, WI, USA) following the manufacturer’s standard protocols (Maxwell® RSC Rumen Microbiome DNA Kit Technical Manual, Promega), including a bead beating step on a FastPrep machine (MP Biomedicals, Irvine, CA, USA) at 4 m/s for 1 min, twice, with a 5 min break between cycles. The V3-V4 region of the 16S rRNA gene was amplified for all bacteria and archaea (sequences provided in Table 1). Thermocycling conditions were: 95oC/5 min: 30 cycles of 98oC/20 s; 55oC/15 s; 72oC/1 min: hold at 4oC (3-step PCR) for bacteria; 95oC/5 min: 30 cycles of 98oC/20 s; 72oC/1 min: hold at 4oC (2-step PCR) for Archaea. In a second PCR, sequencing indexes were added to the amplicons using 96 forward indexes from the Nextera XT Index 1 plate (Illumina, New York, USA) and three reverse indexes (R97, R98, R99), creating 288 unique index combinations. Negative controls were included in both the target amplification and in the indexing PCRs. Library purification, mixing and sequencing followed the Illumina 16S Metagenomic Sequencing Library preparation document (#15044223). Paired-end sequencing (2 × 300 bp) was performed using the MiSeq Reagent Kit v3 (600 cycles, Illumina, New York, USA).

**Table 1:** Primers used to amplify the V3-V4 region of the 16S rRNA gene from bacteria (Bac) and archaea (Arch) (61–63).

Primer name	Target region	Primer sequence (5’–3’)
BacF	16S	GTCTCGTGGGCTCGGAGATGTGTATAAGAGACAGGGACTACHVGGGTWTCTAAT
BacR	16S	TCGTCGGCAGCGTCAGATGTGTATAAGAGACAGCCTACGGGAGGCAGCAG
ArchF	16S	TCGTCGGCAGCGTCAGATGTGTATAAGAGACAGGYGCASCAGKCGMGAAW
ArchR	16S	GTCTCGTGGGCTCGGAGATGTGTATAAGAGACAGHGCYTTCGCCACHGGTRG

### 2.5 Quality control and sequence read counts

A minimum read count of 30,000 for all bacteria and 5,000 for archaea was used to provide a sufficient representation of the taxa present. Those samples that fell below the required sequencing depth (*n* = 12 Bac) were re-sequenced. FastQC (Version0.12.0) was used to assess read quality and determine whether trimming was needed^1^.

### 2.6 Statistical and bioinformatic analysis

Denoising and trimming the raw data was done using the DADA2 plugin (15) within the QIIME2 platform (16). DADA2 was then used to produce representative sequences and amplicon sequence variants (ASV), filtering by sample and feature. ASVs were then merged with metadata for further analysis (feature-table). Final representative sequences were compared against the 16S database, GreenGenes2 (17), using the feature-classifier. Multiple sequence alignment was used to group the sequences with the highest homologies, masking was used to remove errors and ambiguous sequences, and a phylogenetic tree was produced using Fasttree (Version 2.1). MicrobiomeAnalyst v2.0 (18) was used for further analysis, with the feature table, taxonomy, metadata and phylogenetic tree files from QIIME2. Alpha and beta-diversity metrics were calculated to assess the microbial diversity within and between samples over time and with or without the DFM supplement. Alpha-diversity analysis measured the Chao1 (19), Observed (20), and Shannon (21) indices. Beta-diversity analysis, via principal component analysis [PCoA; (22)] and non-metric multidimensional scaling [NMDS; (23)], compared the effect of the DFM supplement at the different experimental time points. The linear discriminant analysis effect size (LEfSe) was used to identify which taxa most likely drove the differences between groups. Both alpha- and beta-diversity and LEfSe were performed at the genus level with statistical significance set at a *p*-value ≤ 0.05.

To test for associations between longitudinal changes in alpha diversity over time and for the different treatment groups, we performed linear mixed-effects (LME) regression analysis for each diversity index. This accounted for subject-specific variation by using cow ID as a random effect while allowing identification of longitudinal differences in alpha/beta diversity due to treatment group by using that category as a fixed effect. The LME models were fitted with a first-order autoregressive correlation. Fitted residuals were assumed to follow a normal distribution with a mean of zero and a variance of σ2. Overall model fit was based on the Akaike information criterion (AIC), Bayesian information criterion (BIC) and visual assessment of Pearson’s residuals against fitted values, Q-Q standardized residuals against standardized normal quantiles violated the normality assumption (24). All analyses used nlme and lme4 (24, 25) statistical packages in R (R team) (26).

Multivariable analysis [MaAsLin2 v1.15.1; (27)] in Rstudio [2024.09.01, Build 394; (28)] was used to quantify the association between ruminal and archaeal taxa and Age, Average, DIM, Month and SUP. Total sum scaling (TSS) normalization and arc-sine square root transformation accounted for instances where abundance was zero in the data. The Benjamini-Hochberg false-discovery method at a threshold of 0.2 was used to adjust the resulting *p*-values (29). The initial analyses were unadjusted, using multiple bivariable models of SUP with the other individual variables. After interpretation of these results, a larger analysis of all variables together was performed. The references used for categorical variables in these analyses were: Experimental group (CON) and Month (Sep 21). Graphical display of the analyses was performed using ggplot2 [v3.5.1; (30)] in Rstudio.

## 3 Results

### 3.1 Bacterial microbiota

The sequencing of the bacterial V3-V4 region of the 16S rRNA gene produced 18,443,231 raw reads, with reads per sample ranging from 32,339 to 233,255 (mean 59,302). For archaea ruminal samples, sequencing generated 3,513,714 raw reads, with reads per sample ranging from 5,011 to 129,376 (mean 8,378). After filtering, the mean number of reads per sample was 52, 004 and 7,948 for total ruminal and archaeal samples, respectively. The number of amplified sequence variants (ASVs) identified was 38,140 for ruminal and 1,186 for archaeal samples. The proportion of Bacillota over time ranged from 33 to 54%, with Bacteroidota ranging from 42 to 50%. Other phyla that comprised a smaller proportion of the ruminal microbiota included Actinomycetota (2.2%), Fibrobacterota (2.2%), Patescibacteria (3.8%), Pseudomonadota (3.6%), Spirochaetota (2.3%) and Verrucomicrobiota (1%). The relative abundance of ruminal microbial communities in CON and DFM samples across different months from April 2021 to June 2023 was graphed for visual display (Supplementary Figure S1). Overall, there are visible differences in the microbial community structure between CON and DFM groups. Over time, some taxa were consistently more abundant in samples from the DFM group than in samples from the CON group.

**Figure 1:**
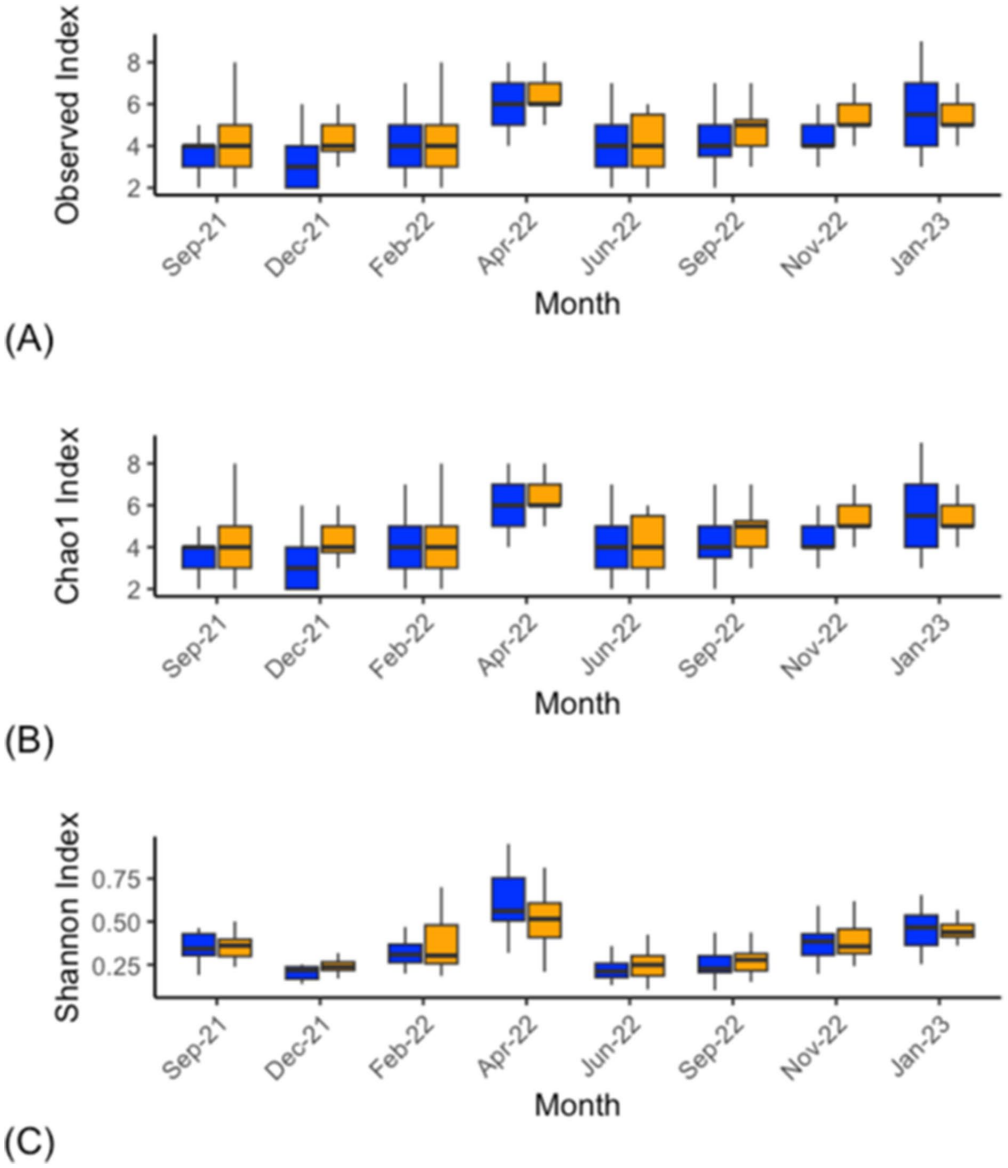
Bacterial alpha-diversity analysis (genus level) within ruminal fluid from CON (blue) and DFM (orange) cows sampled across the study. The Observed **(A)**, Chao1 **(B)**, and Shannon index **(C)**. This indicates that there were significant differences in diversity across the time points. Statistics for individual time points can be found in Supplementary Table S3. The bars across each box represent the median, while the top and bottom whiskers represent the upper and lower quartiles, respectively. Sep-21 is the study baseline.

**Figure 2:**
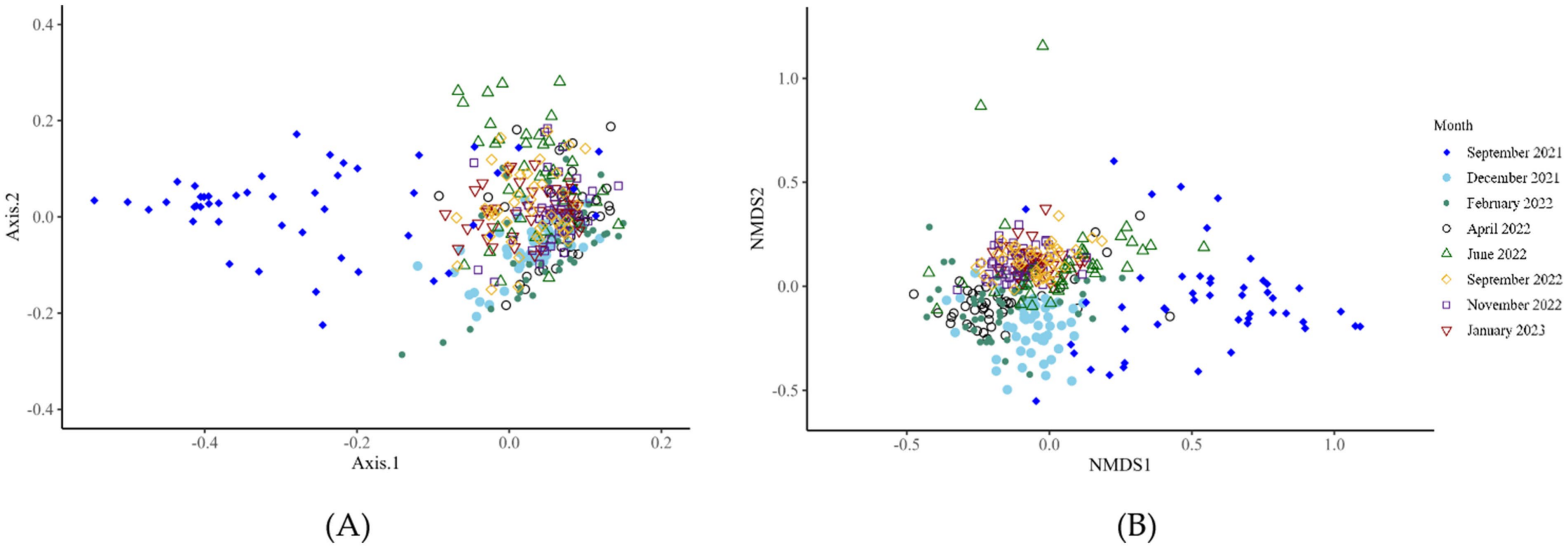
Bacterial beta-diversity (genus level) analysis of ruminal fluid across eight-time points from September 2021 to January 2023. Microbial diversity differed significantly over the 16 months of the trial. Panels **A** and **B**, respectively, display the principal component analysis and non-metric multidimensional scaling plots of the data.

Bacterial alpha-diversity (genus level) differed within the CON and DFM samples for Shannon diversity indices at various time points, including September 2021 (*p* = 0.012), September 2022 (*p* = 0.04) and January 2023 (*p* = 0.01). The other indexes (Observed; *p* > 0.05 and Chao1; *p* > 0.05) did not differ at any time points from September 2021 to January 2023 (Figure 1; Supplementary Table S3). Ruminal bacterial diversity tested at the genus level using beta-diversity analysis differed significantly over the study period across six time points from September 2021 to January 2023 except for April 2022 (*p* = 0.11) and November 2022 (*p* = 0.21) (Figure 2; Supplementary Figure S2; Supplementary Table S4).

**Figure 3:**
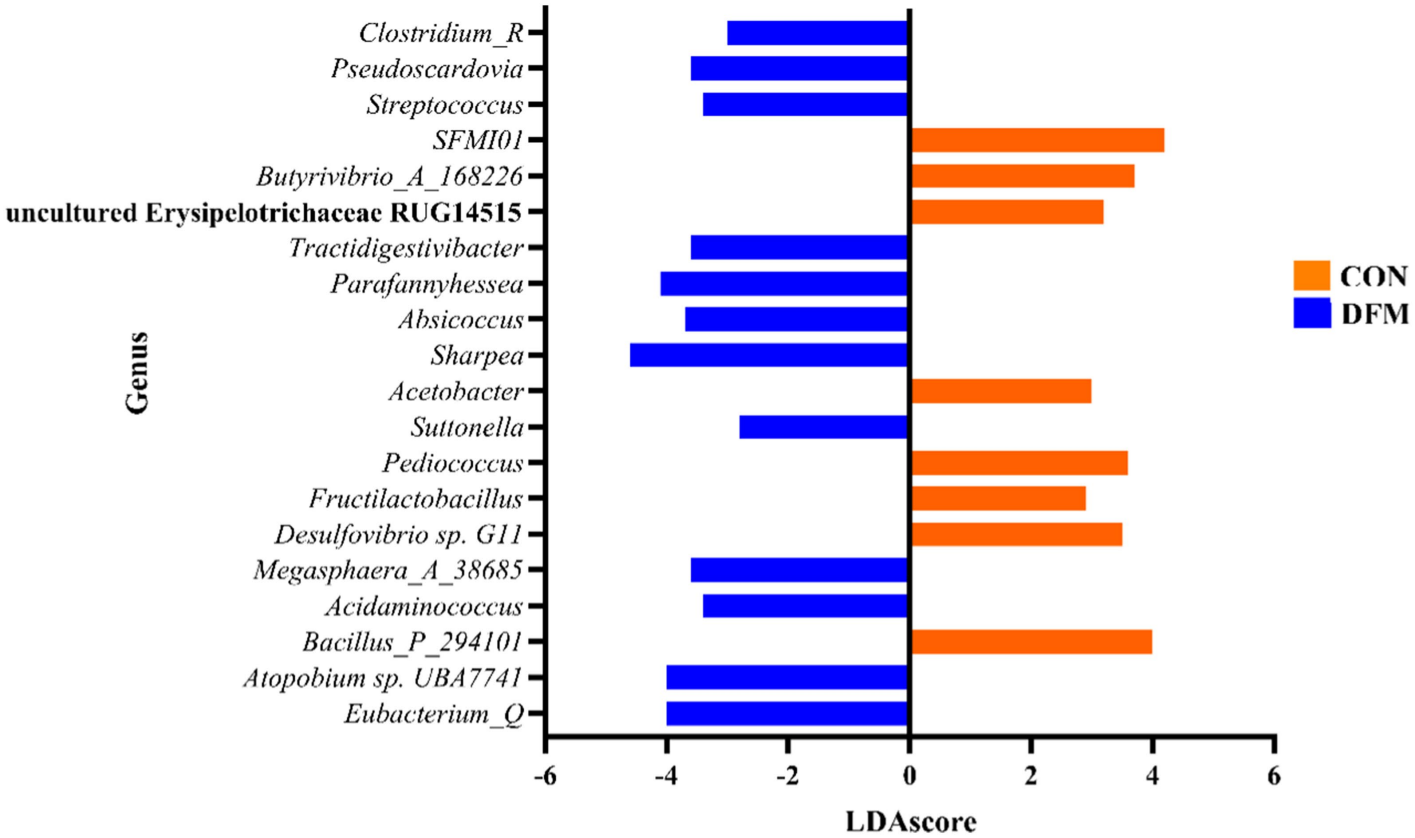
Linear discriminant analysis effect size (LEfSe) analysis of total data from ruminal fluid in CON compared with DFM cows. LDAscore is the linear discriminant analysis effect size score, significant at a *p*-value of < 0.05 and a false discovery rate of <0.2. Total data at each time point is available in Supplementary file 2.

Figure 3 presents the significant differences in the abundance of various genera between the CON and DFM groups. Genera such as *Sharpea, Eubacterium_Q*, *UBA7741*, *Parafannyhessea,* and *Pseudoscardovia* are significantly more abundant in the DFM group. At the same time, *Bacillus_P_294101, SFMI01*, *Butyrivibro_A_168226*, *Pediococcus*, and *G11* show higher abundance in the CON group with positive LDAscores. The results highlight significant shifts in microbial community structure between the CON and DFM groups, with several genera showing marked differences in abundance (*p* ≤ 0.05).

### 3.2 Archaeal microbiota

The abundance data for pooled CON and DFM samples over time identified three families, Methanobacteriaceae (99.3%), Methanomethylophilaceae (0.69%) and Methanosarcinaceae (0.001%) within the Methanobacteriota.

**Figure 4:**
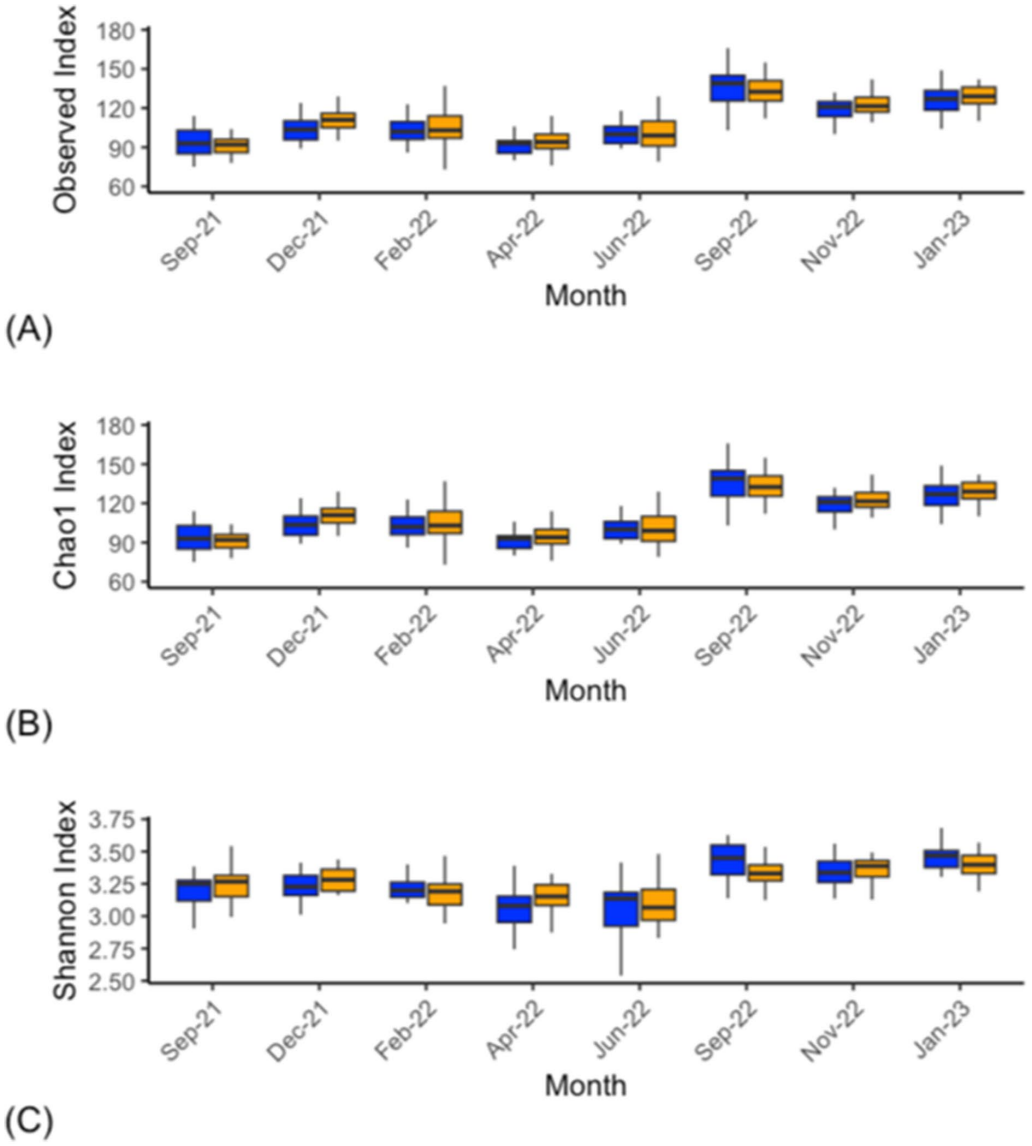
Alpha diversity analysis (genus level) within the archaea of ruminal fluid of CON (blue) and DFM (orange) cows. Observed **(A)**, Chao1 **(B)** and Shannon index **(C)**. For individual time point statistics, refer to Supplementary Table S5. The bars across each box represent the median, while the top and bottom whiskers represent the upper and lower quartiles. Sep-21 is the study baseline.

The archaeal alpha-diversity analysis at the genus level from the CON and DFM cows over various time points demonstrated significant differences in the Observed and Chao1 indices in the September 2021 (*p* ≤ 0.01), April 2022 (*p* ≤ 0.01) and January 2023 (*p* ≤ 0.015) samples. Significant differences in September 2021 (*p* ≤ 0.01), December 2021 (*p* ≤ 0.01), and April to September 2022 (*p* ≤ 0.01) were observed for the Shannon index (Figure 4; Supplementary Table S5).

**Figure 5:**
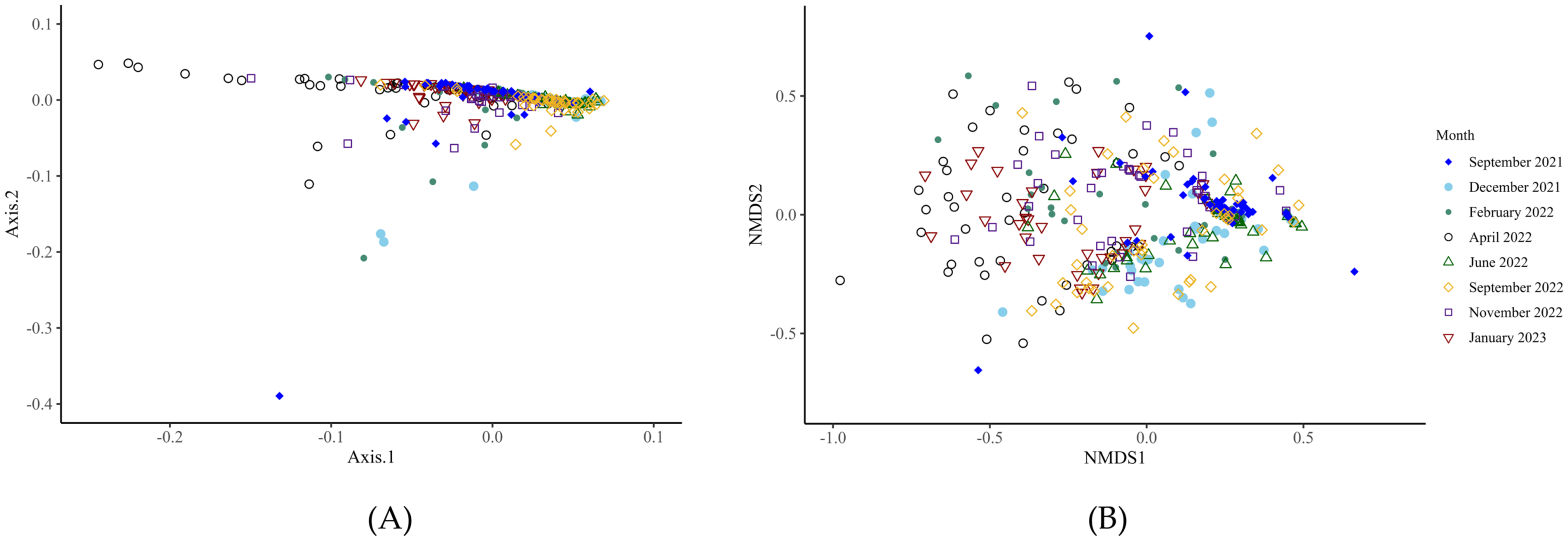
Beta-diversity analysis (genus level) of the archaea of ruminal fluid over the time points investigated. *F*-value: 17.7; R2: 0.3; *p*-value: 0.001; Stress: 0.2. The microbial diversity of the archaea changes significantly over time. *F*-value: 17.73, R2: 0.27, *p*-value: 0.001, Stress, 0.16. Panels **A** and **B**, respectively, display the principal component analysis and non-metric multidimensional scaling plots of the data.

The beta-diversity analysis at the genus level of archaea identified no significant differences (*p* > 0.05) at any time point between the CON and DFM cows (Figure 5; Supplementary Table S6, Supplementary Figure S3). The beta-diversity did significantly vary over time.

### 3.3 Multivariate analysis

**Figure 6:**
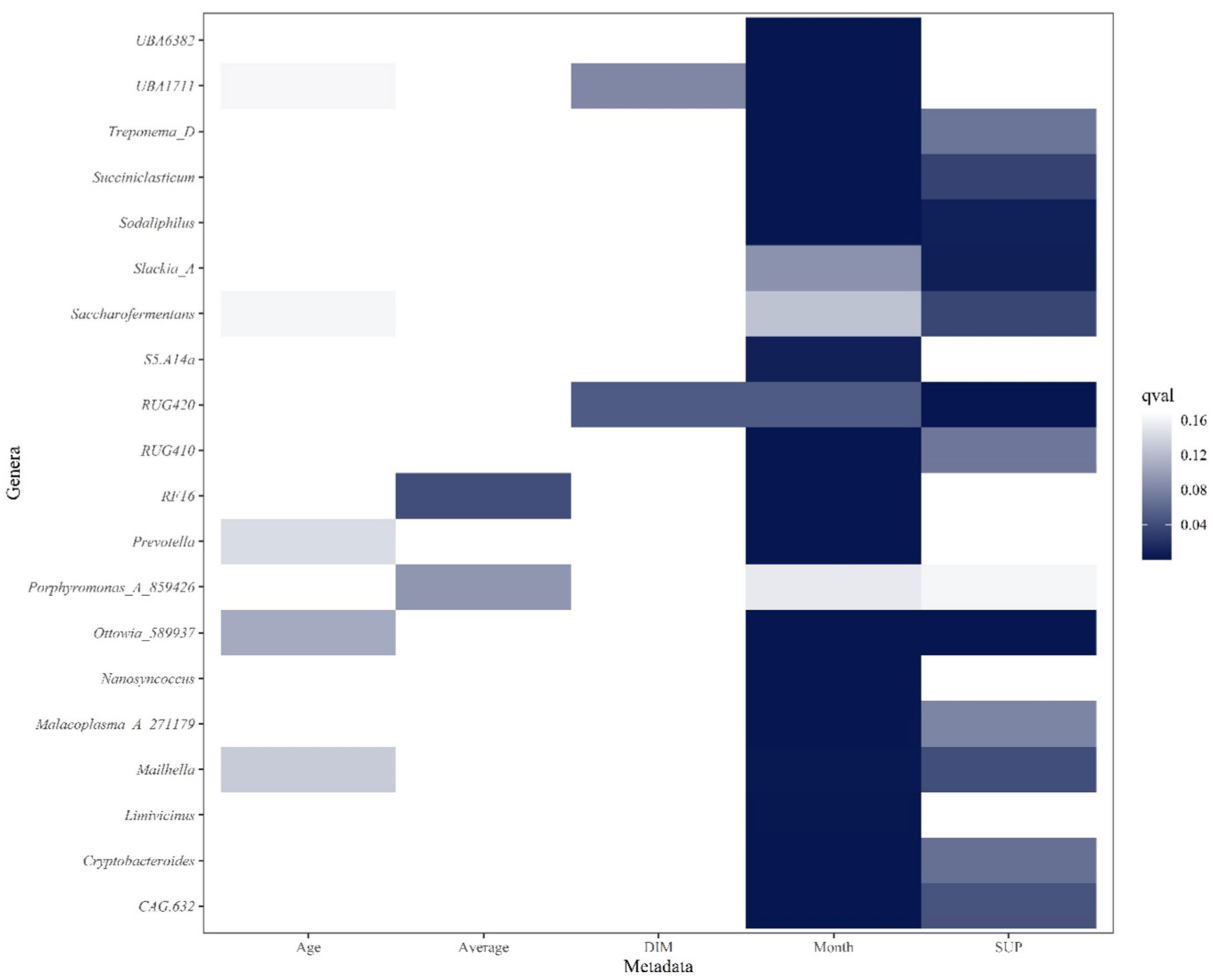
Heatmap of top 20 ruminal genera that are significantly associated with Age (years), Average milk (l/day), days in milk (DIM), calendar month (Month) and experimental group (SUP). Significant interactions (*p* ≤ 0.05, FDR < 0.2) are colored in different shadings of blue, with the most intense being the most significant. Genera are in reverse alphabetical order.

**Figure 7:**
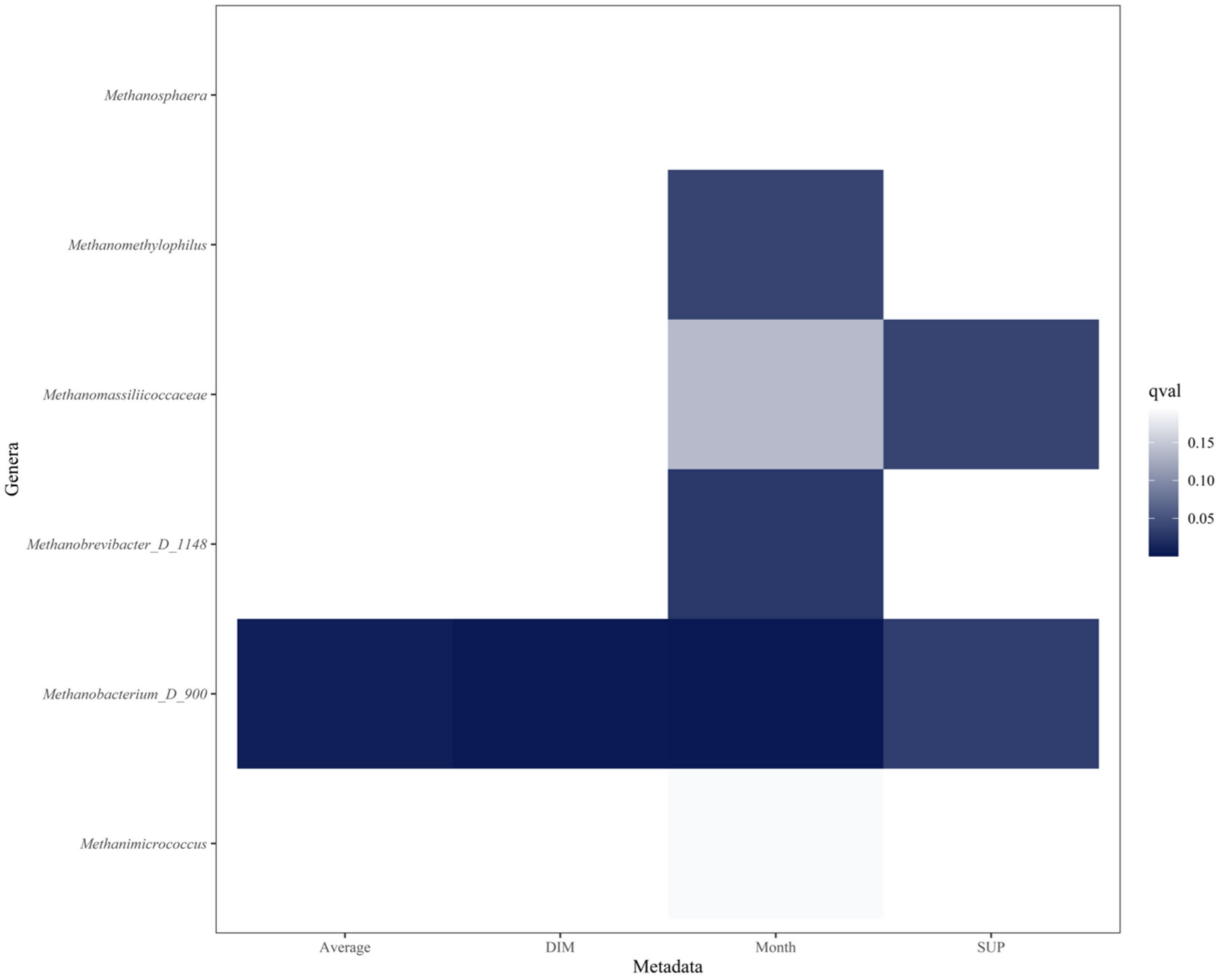
Heatmap of archaeal genera that are significantly associated with Age (years), Average milk (l/day), days in milk (DIM), calendar month (Month) and experimental group (SUP). Significant interactions (*p* ≤ 0.05, FDR < 0.2) are colored in different shadings of blue, with the most intense being the most significant. Genera are in reverse alphabetical order. No archaeal genera were significantly associated with age.

The graph displays the top 20 genera from the multivariable analysis of ruminal fluid and the five productivity measures investigated (Figures 6, 7). The overall multivariate analysis is presented in the Supplementary Figures S4–S8.

## 4 Discussion

In the present study, Firmicutes and Bacteroidetes were the predominant phyla in the rumen fluid samples of dairy cows, with other less prevalent phyla, including Actinomycetota, Fibrobacterota, Patescibacteria, Pseudomonadota, Spirochaetota, and Verrucomicrobiota. These findings are consistent with previous studies (3, 31). *Prevotella* was the predominant genus (29%) within the phylum Bacteroidota in the rumen, which is crucial for protein degradation and starch utilization (32). Henderson et al. (33) noted that while the microbiota of ruminants from different geographic regions varied, a core microbiota could be identified. This core microbiota included the seven most abundant bacterial groups, including *Prevotella*, *Butyrivibrio*, *Ruminococcus*, and unclassified members of *Lachnospiraceae*, *Ruminococcaceae*, *Bacteroidales*, and *Clostridiales*. *Proteobacteria* have been recognized as part of the rumen core microbiota, as demonstrated by Seshadri et al. (34), Stewart et al. (35), and Stewart et al. (36), highlighting the growing understanding of microbial diversity and the crucial role of advanced sequencing technologies in reshaping our knowledge of the rumen ecosystem. These groups of core microbiota were present in all samples regardless of species, diet, or geographical location but did vary in abundance. Their study encompassed a variety of ruminant species, including cattle, buffalo, bison, sheep, goats, deer, giraffes, and camelids.

The dominance of Bacillota and Bacteroidota among rumen microbiota sampled in this study is consistent with previous studies (37). Temporal variations observed in the current study indicated that external factors, such as diet, environment, and management practices, significantly influence microbial communities (38). Additionally, the lack of significant differences in alpha diversity between DFM-supplemented and control cows indicates that DFM supplementation does not drastically alter overall microbial diversity (39). This observation is consistent with other studies examining the effect of feed additives like DFMs on ruminal microbiota in which subtle changes are typical rather than any dramatic shifts in microbial diversity (40). This indicates that the supplement impact is nuanced and more likely to affect specific taxa rather than the entire microbial community (41, 42). Beta-diversity analysis and LEfSe results suggest that the DFM supplement influenced specific microbial taxa (43, 44), supporting the conclusion that while overall diversity remained stable, the supplement altered the abundance of certain bacteria. Such changes in microbial composition could impact the cows’ fermentation processes and nutrient absorption. Consequently, optimizing the digestive processes has the potential to improve cattle health and productivity. Finally, the beta-diversity analysis and LEfSe results indicate that the DFM supplement influences the composition of specific microbial taxa within the rumen, as demonstrated by genera like *Prevotella*, which had significant changes in abundance. The result aligns with prior research suggesting that DFM selectively promote or inhibits specific microbial populations, thereby impacting the overall fermentation process (45).

The abundance of the lactic acid-producing genera decreased as animals transitioned to a more starch-based diet, likely due to a reduction in its key substrates, such as amylopectin, maltotriose, and maltodextrin, which are needed for lactic acid production (46). *Methanobrevibacter* is uniquely adapted to the rumen environment as an obligate anaerobe, producing methane as its primary metabolic byproduct (47). This study’s observed shifts in microbial community composition likely reflect the influence of DFM and rumen conditions on microbial dynamics.

Recent studies have supported similar effects in response to various dietary interventions and feed additives (48, 49). These outcomes underline the dynamic responses of microbial communities to external interventions and bring to focus the risk of significant implications for animal health (enhanced nutrient absorption and immune responses). This emphasizes the importance of comprehending and optimizing the rumen microbial balance in ruminant nutrition (7). The observed changes in specific microbial taxa in this study, such as the increase in *Prevotella* abundance among DFM-supplemented cows, may carry significant functional implications with the potential to influence enhancing complex carbohydrate degradation and improve nutrient absorption (32, 50). Similarly, alterations in the abundance of other key genera (such as *Ruminococcus* and *Succiniclasticum*) could have implications for the breakdown of fiber and the production of short-chain fatty acids crucial to the energy metabolism of dairy cows (51, 52). The functional significance of other bacterial genera that were associated with DFM supplementation, such as *Treponema, Succiniclasticum, Saccharofermentans* and *Ottawia* have been identified. These genera are known to have a significant role in fiber degradation, fatty acid production and nutrient metabolism and therefore likely play an important role feed efficiency and productivity (53). The functional significance of the many other genera found in the present study to be significantly associated with DFM supplementation and/or bovine productivity remains to be elucidated. The findings underline the intricate complexity of the interaction between microbial composition and metabolic processes in the rumen and serve further to highlight the importance of understanding and optimizing microbial communities to achieve enhanced animal performance (54, 55).

Archaea constituted approximately 2.5% of the ruminal microbiota (56), higher than that observed in the present study. Previous research has indicated that the bacterial community in the rumen is taxonomically richer than the ruminal archaea, reflecting a pattern of similar and limited diversity of the archaeal populations when compared to bacteria, also found in the current study (33). The archaeal microbiota, particularly members of the Methanobacteriaceae family, play a pivotal role in ruminal fermentation by driving methanogenesis, which balances hydrogen levels but contributes to energy losses and greenhouse gas emissions (57). Exploring the impact of DFM supplementation on archaeal populations is essential, as DFMs may alter substrate availability or interspecies interactions, potentially mitigating methane production and improving rumen efficiency (58).

The temporal changes observed are crucial to understanding the adaptability and resilience of the microbial community in response to external interventions, like the DFM supplement. This study unveiled significant temporal variations in microbial diversity, consistent with previous research. These fluctuations suggest that the relative abundances of the core rumen and ruminal microbial groups fluctuate over time, in turn, potentially affecting the fermentation efficiency and overall health of cattle (59, 60). Therefore, continuous monitoring and effective management practices are required to optimize rumen microbial balance. Comparative studies across various breeds, diets, and geographic regions would provide a more comprehensive understanding of the core microbiota and the drivers of the fluctuations. Valuable insights into the factors influencing microbial dynamics in dairy cows may be discovered, allowing improved tailoring of management practices that optimize animal health and productivity compatible with local breed(s), geography, ration ingredients, and other inputs. The findings of this study offer valuable insights into how the DFM supplement often affects the microbiota composition and diversity in dairy cows. There are significant potential implications for animal health and productivity due to improved knowledge of the complexity of the ruminal microbiota. All this serves to emphasize the potential role of DFMs in the planned and controlled modulation of microbial communities in livestock.

To advance this study’s findings, future research should prioritize longitudinal studies investigating the persistence of any effects of the DFM supplement on microbial community structure and function and long-term effects on dairy cows. Employing metagenomic and metabolomic approaches for functional analysis would offer deeper insights into the functional roles of the affected microbial taxa. Comparative studies across diverse breeds, diets, and geographic locations would facilitate a comprehensive understanding of the core ruminal microbiota and its variations, elucidating broader patterns and influences. Furthermore, the study focused on temporal variations in microbial diversity, but the study design did not account for potential seasonal effects or diet changes during the 18-month period that could influence microbial composition. While 16S rRNA sequencing provides insights into microbial taxonomy, it does not capture functional activities or metabolic pathways, limiting understanding of how microbial shifts impact cow health and production. Furthermore, not all microbial communities were identified using current databases. Expanding microbial databases and improving taxonomic classification methods are essential for accurately characterizing microbial communities and their functional roles. In particular, upgrading bacterial and archaeal databases is crucial for better understanding fungal microbiota. Collectively, these efforts would enhance our understanding of how DFM supplementation shapes microbial communities, influences functional dynamics, and informs targeted strategies to optimize animal health, productivity, and welfare in cattle production systems. Finally, the results of this study can only be extrapolated to dairy herds similar to the study animals with similar settings. Although the study has strong internal validity, the external validity is limited. Therefore, the results cannot be generalized to the larger dairy cow populations.

## 5 Conclusion

This study demonstrated that a lactobacilli-based DFM supplement over an extended period alters the composition and diversity of the ruminal microbiota in dairy cows. The temporal change of ruminal microbiota was mainly explained by calendar month, highlighting the dynamic nature and potentially other factors that influenced temporality that were not accounted for in this study. Future research using metagenomic and metabolomic approaches and comparative studies across different breeds, diets, and geographic locations is recommended for a better understanding of the impact of the DFM supplement.

## Data Availability

The original contributions presented in the study are included in the article/Supplementary material, further inquiries can be directed to the corresponding authors.

## Footnotes

^1^ http://www.bioinformatics.babraham.ac.uk/projects/fastqc/
