# Supplementary material for: Temporal changes in ruminal microbiota composition and diversity in dairy cows supplemented with a lactobacilli-based DFM
Source: Front Vet Sci. 2025 May 16;12:1584959. doi: 10.3389/fvets.2025.1584959 (PMC12123876; doi:10.3389/fvets.2025.1584959)
Supplement: Supplementary file 1 [file Data_Sheet_1.pdf]

Table S1: Ingredients and chemical composition of the diet (%, DFM basis).

**Ingredient**	**Mean (%)**	**Min**	**Max**
Grain (barley, wheat, sorghum)	19.3	15.8	27.7
Protein Meal (canola meal, soybean meal)	7.7	0	17.4
Byproducts (flour bread, carrots, sweet corn waste, chickpea millrun)	14.3	3.1	35.3
Lucerne Hay	5.8	3.3	9.9
Silage (corn, barley, oats, soybean)	34.7	14.8	49.2
Pasture (kikuyu, ryegrass)	15.3	0	43
Bypass Fat	0.6	0	1.9
Minerals (macro minerals, trace minerals premix, urea, mycotoxin binder)	2.4	1.2	3.5
**Diet Composition**			
Crude Protein (CP, %)	16.9	13.8	22.8
Neutral Detergent Fiber (NDF, %)	35.9	31.2	40
Acid Detergent Fiber (ADF, %)	23.2	19.3	26.2
Non-Fibrous Carbohydrate (NFC, %)	34.3	28.1	40.2
Fat (%)	4.4	3.6	5.6
MJ ME/Kg DM	9.5	8.6	10.8
Starch (%)	23	20.1	26.5

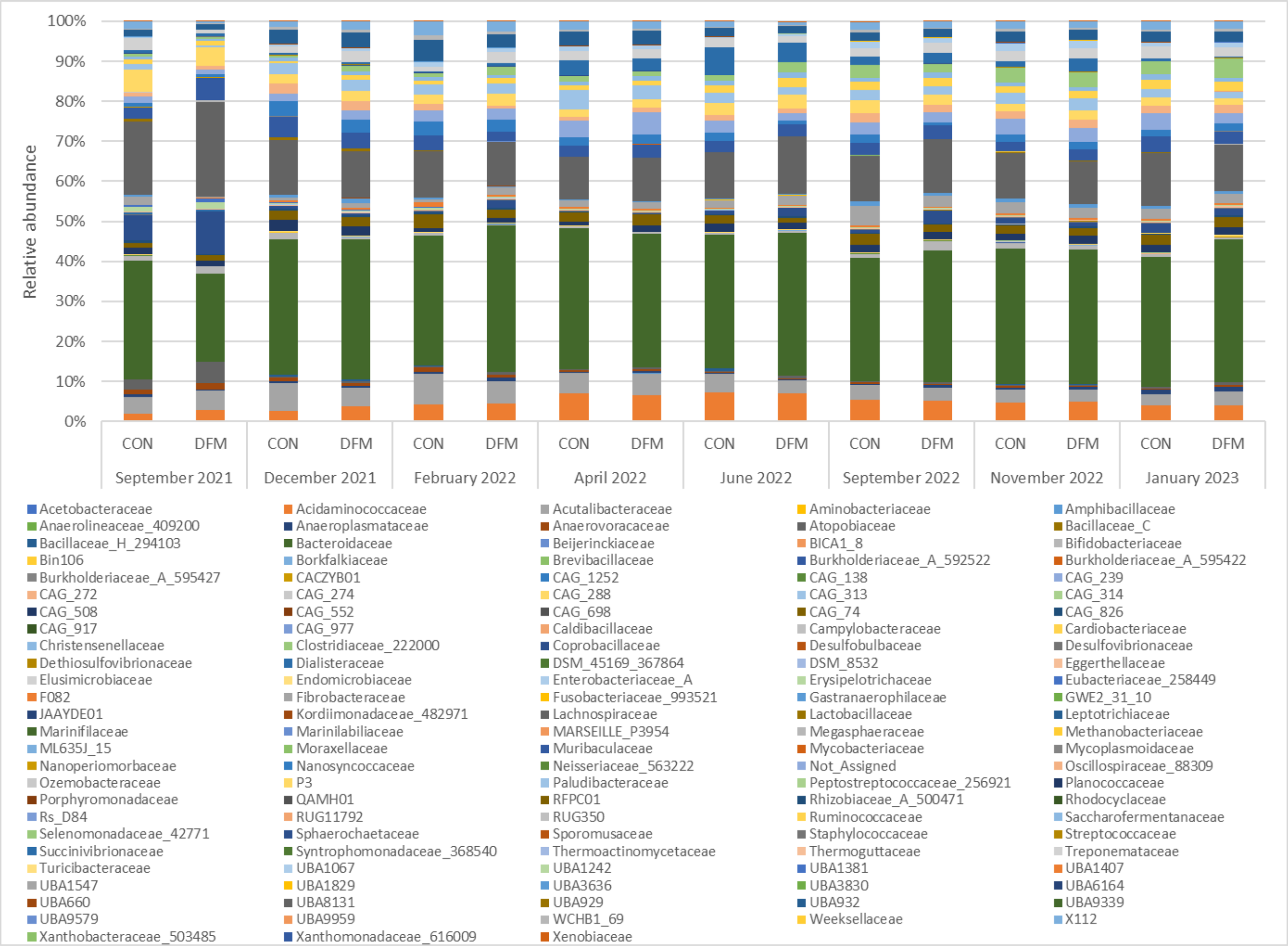

Figure S1: Relative abundance of bacterial families in the rumen of Control (CON) and DFM-supplemented (DFM) cows over the duration of the study.

Table S2: Descriptive statistics of cow records and milk yield, milk component, and liveweight (LW) measurements stratified by experimental groups.

	**Mean (SD)**	**Median (Q1–Q3)**	**Min–Max**	**Mean (SD)**	**Median (Q1–Q3)**	**Min–Max**
**Baseline (before the start of the study period)**						
Milk yield (L)	33 (7)	33 (27–38)	19, 50	32 (8)	31 (26–37)	11, 49
Days in milk (days)	131 (53)	145 (87–172)	6, 226	135 (54)	146 (88–174)	5, 423
Parity	2 (1)	2 (1–3)	1, 3	2 (1)	2 (1–3)	1, 3
Fat yield (kg)	3 (1)	3 (3–4)	2, 10	3 (1)	3 (3–4)	2, 7
Protein yield (kg)	3 (0)	3 (3–3)	3, 4	3 (0)	3 (3–3)	3, 5
Somatic cell counts (x1000 cells/mL)	70 (73)	39 (20–93)	8, 356	98 (92)	61 (31–139)	9, 354
Liveweight (kg)	582 (57)	584 (534–612)	465, 707	577 (62)	577 (524–622)	437, 733

Table S3: Bacterial alpha-diversity indices within ruminal fluid from dairy cows supplemented with DFM compared to Control cows over the study period.

Alpha diversity index	Variable	Group		SEM	P-value		
		CON	DFM		Group	Month	Group×Month
Observed	Sep-21	94	91	2.24	0.43	<0.01	0.41
	Dec-21	104	111	1.94		<0.01	0.05
	Feb-22	103	105	3.09		<0.01	0.31
	Apr-22	92	96	2.26		0.50	0.17
	Jun-22	98	100	3.05		0.25	0.29
	Sep-22	136	133	3.16		<0.01	0.87
	Nov-22	119	123	2.33		<0.01	0.15
	Jan-23	126	129	3.53		<0.01	0.25
Chao1	Sep-21	94	91	2.24	0.43	<0.01	0.41
	Dec-21	104	111	1.94		<0.01	0.05
	Feb-22	103	105	3.09		0.01	0.31
	Apr-22	92	96	2.26		0.50	0.17
	Jun-22	98	100	3.05		0.25	0.29
	Sep-22	136	133	3.16		<0.01	0.87
	Nov-22	119	123	2.33		<0.01	0.15
	Jan-23	126	129	3.53		<0.01	0.25
Shannon	Sep-21	3.21	3.24	0.04	0.50	<0.01	0.01
	Dec-21	3.22	3.29	0.03		0.50	0.53
	Feb-22	3.22	3.17	0.03		0.86	0.19
	Apr-22	3.06	3.17	0.04		0.84	0.19
	Jun-22	3.07	3.09	0.04		<0.01	0.81
	Sep-22	3.43	3.33	0.03		<0.01	0.04
	Nov-22	3.34	3.36	0.03		<0.01	0.83
	Jan-23	3.46	3.38	0.03		<0.01	0.01

Key: CON Control; DFM Direct Fed Microbial; SEM largest Standard Error of the Mean; P-values are Wald test P-values derived from a mixed effects linear model with an autoregressive correlation structure of the error terms, cow fitted as a random term and Group X Month fitted as a fixed term.

Table S4: Bacterial beta-diversity analysis (genus level) of the microbial diversity of ruminal fluid from supplemented and control cows over the eight sampling time points. A p-value ≤ 0.05 is considered significant.

	**F-value**	**R^2**	**p-value**	**Stress**
September 2021	10	0.18	0.001	0.15
December 2021	6.3	0.12	0.001	0.27
February 2022	6.3	0.13	0.001	0.21
April 2022	1.6	0.04	0.11	0.24
June 2022	2.9	0.07	0.03	0.14
September 2022	6.4	0.13	0.001	0.22
November 2022	1.3	0.03	0.21	0.25
January 2023	6.1	0.13	0.001	0.27

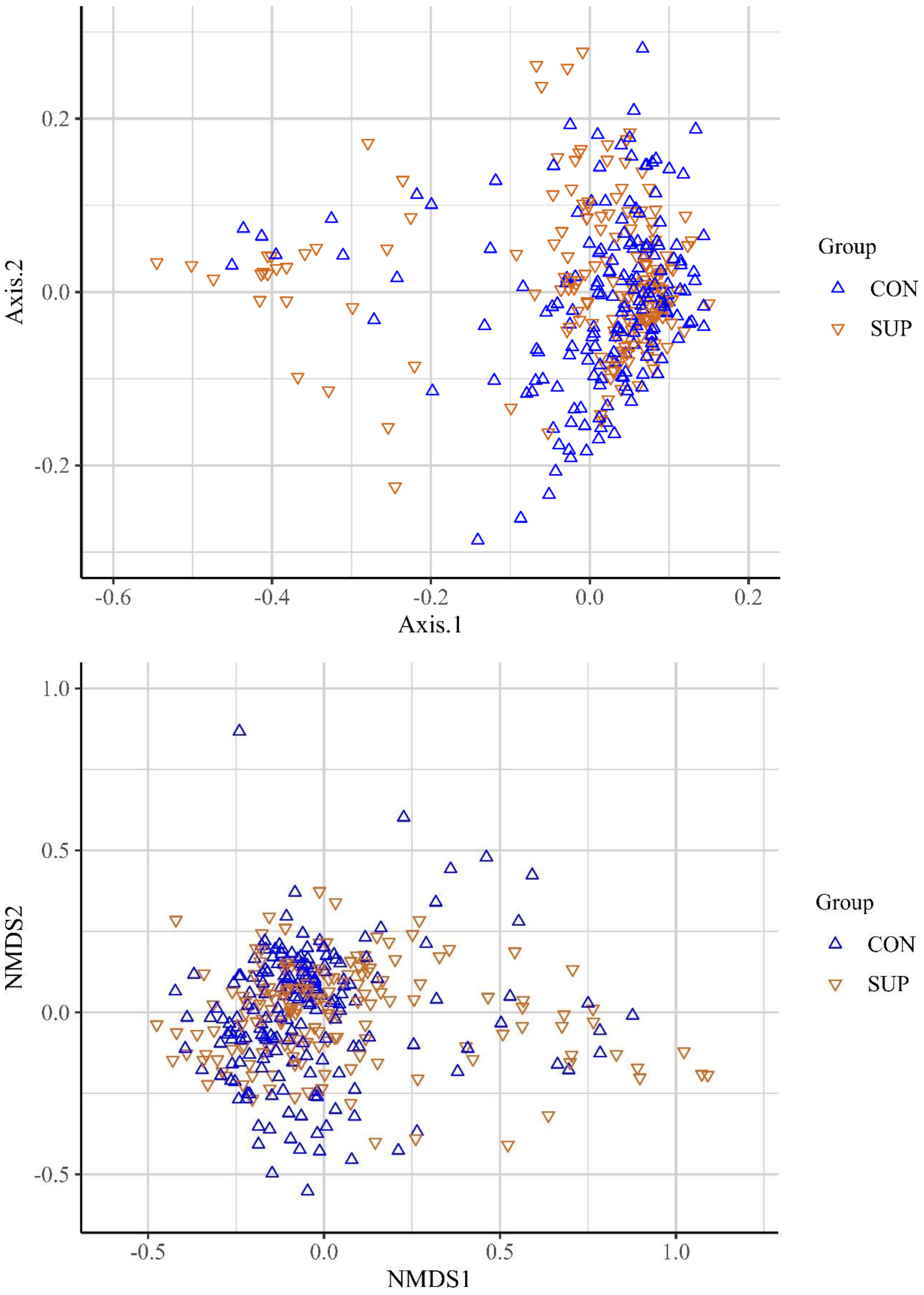

Figure S2: Bacterial beta-diversity analysis (genus level) of ruminal fluid from DFM supplemented (SUP) and control (CON) cows. F-value: 4.324; R^2: 0.012; p-value: 0.001*; Stress: 0.183. Supplementation had a significant effect on the microbial diversity of ruminal fluid.

Table S5: Archaea alpha-diversity indices within ruminal fluid from dairy cows supplemented with DFM compared to Control cows over the study period.

Alpha diversity index	Variable	Group		SEM	P-value		
		CON	DFM		Group	Month	Group×Month
Observed	Sep-21	3.61	4.29	0.32	0.09	<0.01	0.18
	Dec-21	3.21	4.42	0.25		0.34	0.31
	Feb-22	4.04	4.33	0.35		0.30	0.53
	Apr-22	6.17	6.05	0.25		<0.01	0.16
	Jun-22	3.81	4.26	0.29		0.82	0.94
	Sep-22	4.22	4.85	0.26		0.24	0.81
	Nov-22	4.74	5.37	0.33		0.01	0.99
	Jan-23	5.59	5.47	0.36		<0.01	0.17
Chao1	Sep-21	3.61	4.29	0.32	0.09	<0.01	0.18
	Dec-21	3.21	4.42	0.25		0.34	0.31
	Feb-22	4.04	4.33	0.35		0.30	0.53
	Apr-22	6.17	6.05	0.25		<0.01	0.16
	Jun-22	3.81	4.26	0.29		0.82	0.94
	Sep-22	4.22	4.85	0.26		0.24	0.81
	Nov-22	4.74	5.37	0.33		0.01	0.99
	Jan-23	5.59	5.47	0.36		<0.01	0.17
Shannon	Sep-21	0.39	0.36	0.03	0.31	<0.01	0.07
	Dec-21	0.25	0.24	0.03		<0.01	0.72
	Feb-22	0.34	0.37	0.03		0.21	0.22
	Apr-22	0.61	0.52	0.04		<0.01	0.27
	Jun-22	0.22	0.25	0.02		<0.01	0.11
	Sep-22	0.25	0.3	0.03		<0.01	0.05
	Nov-22	0.38	0.4	0.03		0.82	0.29
	Jan-23	0.46	0.45	0.02		0.06	0.70

Key: CON Control; DFM Direct Fed Microbial; SEM largest Standard Error of the Mean; P-values are Wald test P-values derived from a mixed effects linear model with an autoregressive correlation structure of the error terms, cow fitted as a random term and Group X Month fitted as a fixed term.

Table S6: Beta-diversity analysis (genus level) of the archaea of ruminal fluid in control compared with DFM-supplemented cows over the time points tested.

	**F-value**	**R^2**	**p-value**	**Stress**
September 2021	0.7	0.02	0.7	0.09
December 2021	2.8	0.06	0.1	0.13
February 2022	0.3	0.01	0.8	0.12
April 2022	2.7	0.06	0.1	0.22
June 2022	0.4	0.01	0.7	0.08
September 2022	2.5	0.06	0.1	0.15
November 2022	0.8	0.02	0.5	0.15
January 2023	0.9	0.02	0.4	0.14

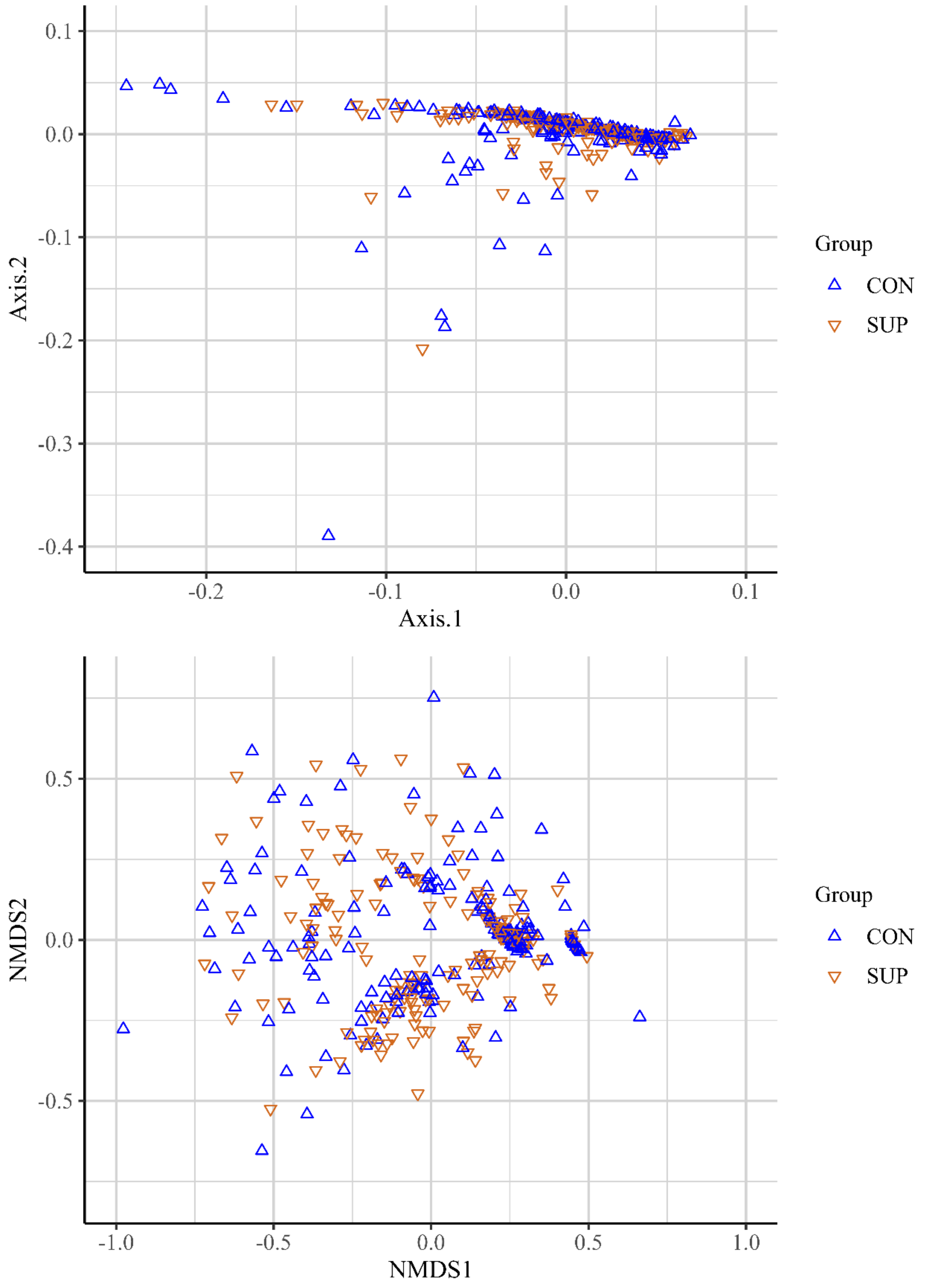

Figure S3: Beta-diversity analysis (genus level) of the archaea of ruminal fluid in control (CON) compared with DFM-supplemented (SUP) cows. F-value: 1.007; R^2: 0.0029; p-value: 0.367; Stress: 0.156. The microbial diversity of the archaea is not significantly influenced by the treatment regime.

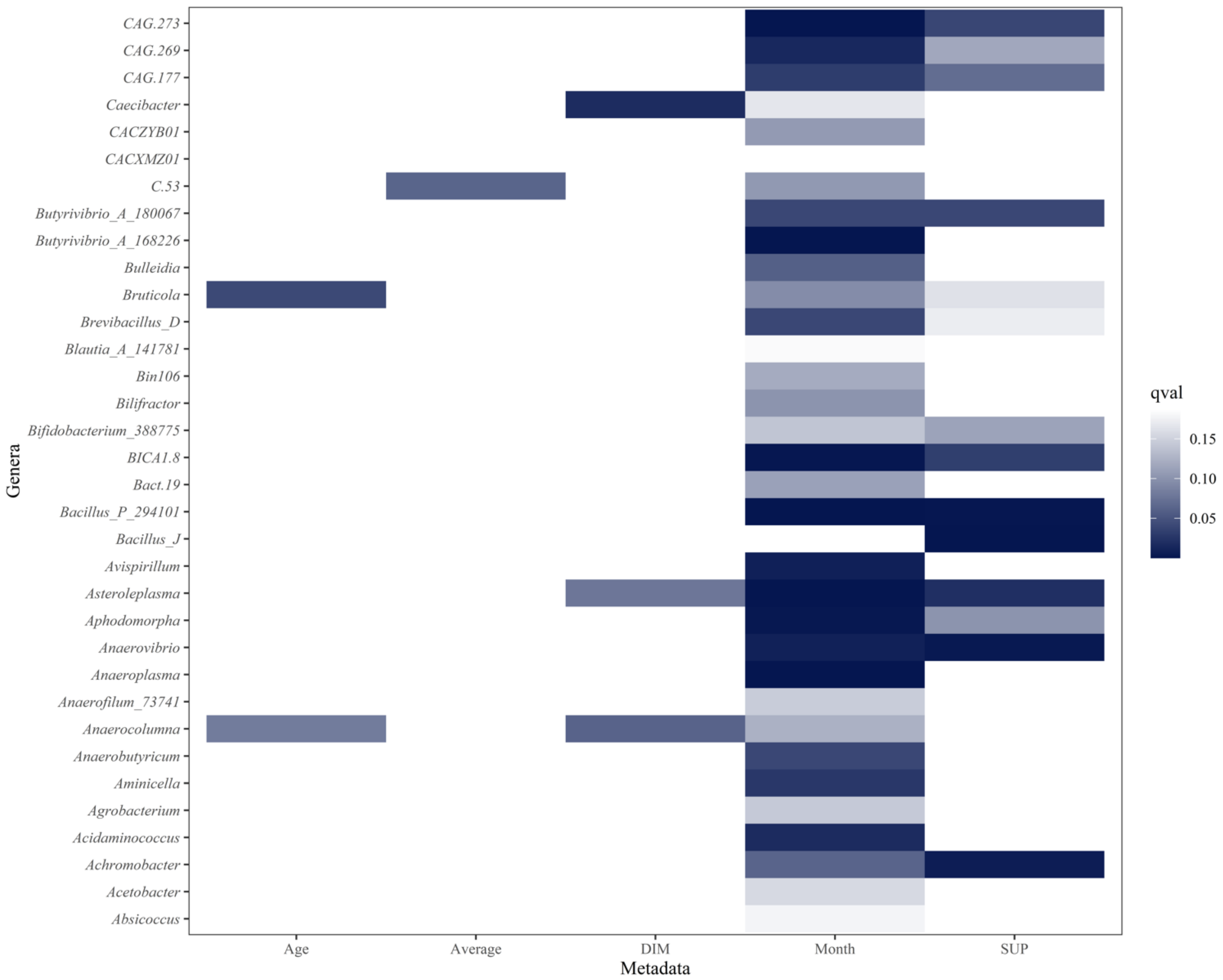

Figure S4: Heatmap of ruminal genera that are significantly associated with Age (years), Average milk (l/day), days in milk (DIM), calendar month (Month) and experimental group (SUP). Significant interactions ($P \leq 0.05$, FDR < 0.2) are colored in different shadings of red, with the most intense being the most significant. Genera are in reverse alphabetical order.

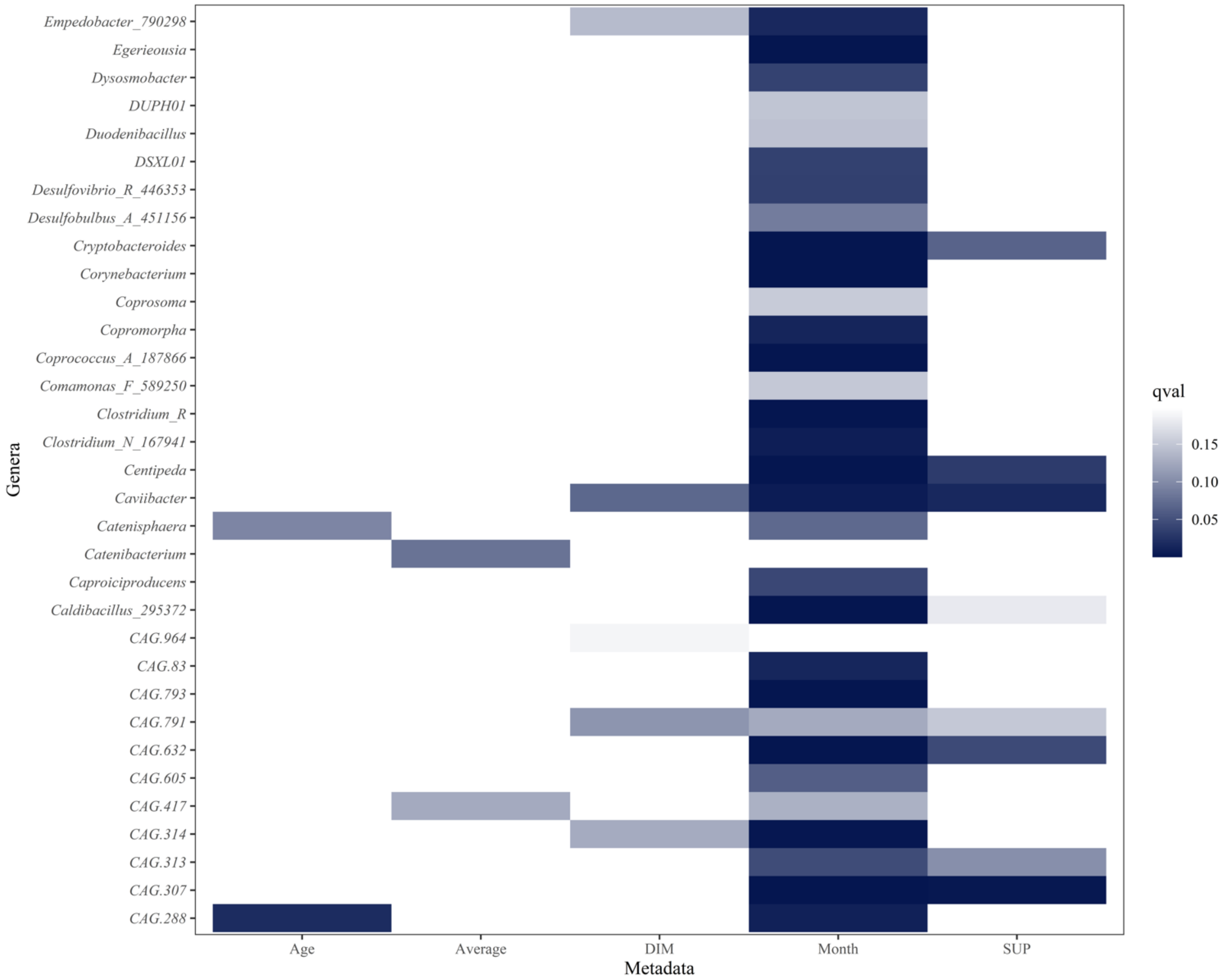

Figure S5: Heatmap of ruminal genera that are significantly associated with Age (years), Average milk (l/day), days in milk (DIM), calendar month (Month) and experimental group (SUP). Significant interactions ($P \leq 0.05$, FDR < 0.2) are colored in different shadings of red, with the most intense being the most significant. Genera are in reverse alphabetical order.

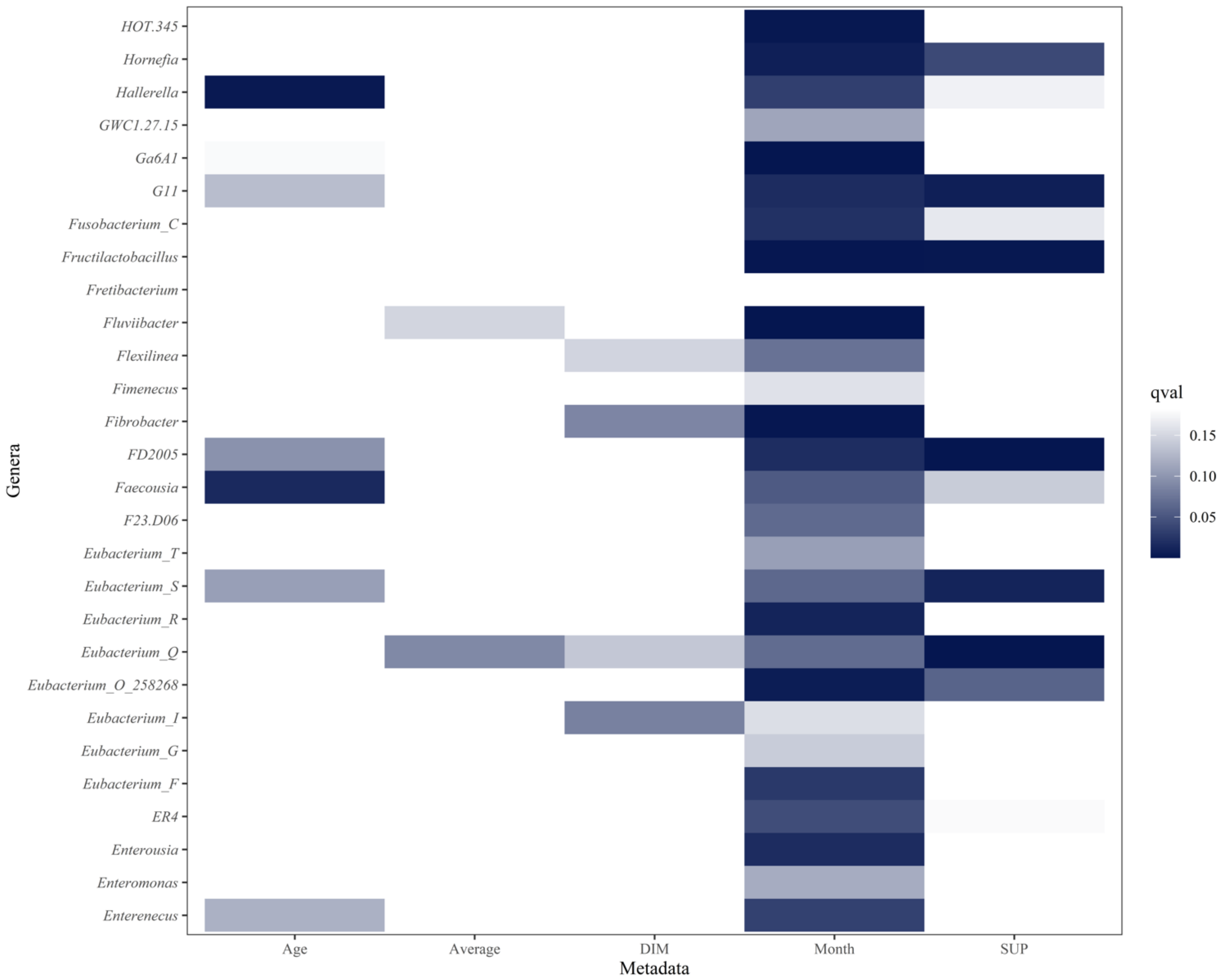

Figure S6: Heatmap of ruminal genera that are significantly associated with Age (years), Average milk (l/day), days in milk (DIM), calendar month (Month) and experimental group (SUP). Significant interactions ($P \leq 0.05$, $FDR < 0.2$) are colored in different shadings of red, with the most intense being the most significant. Genera are in reverse alphabetical order.

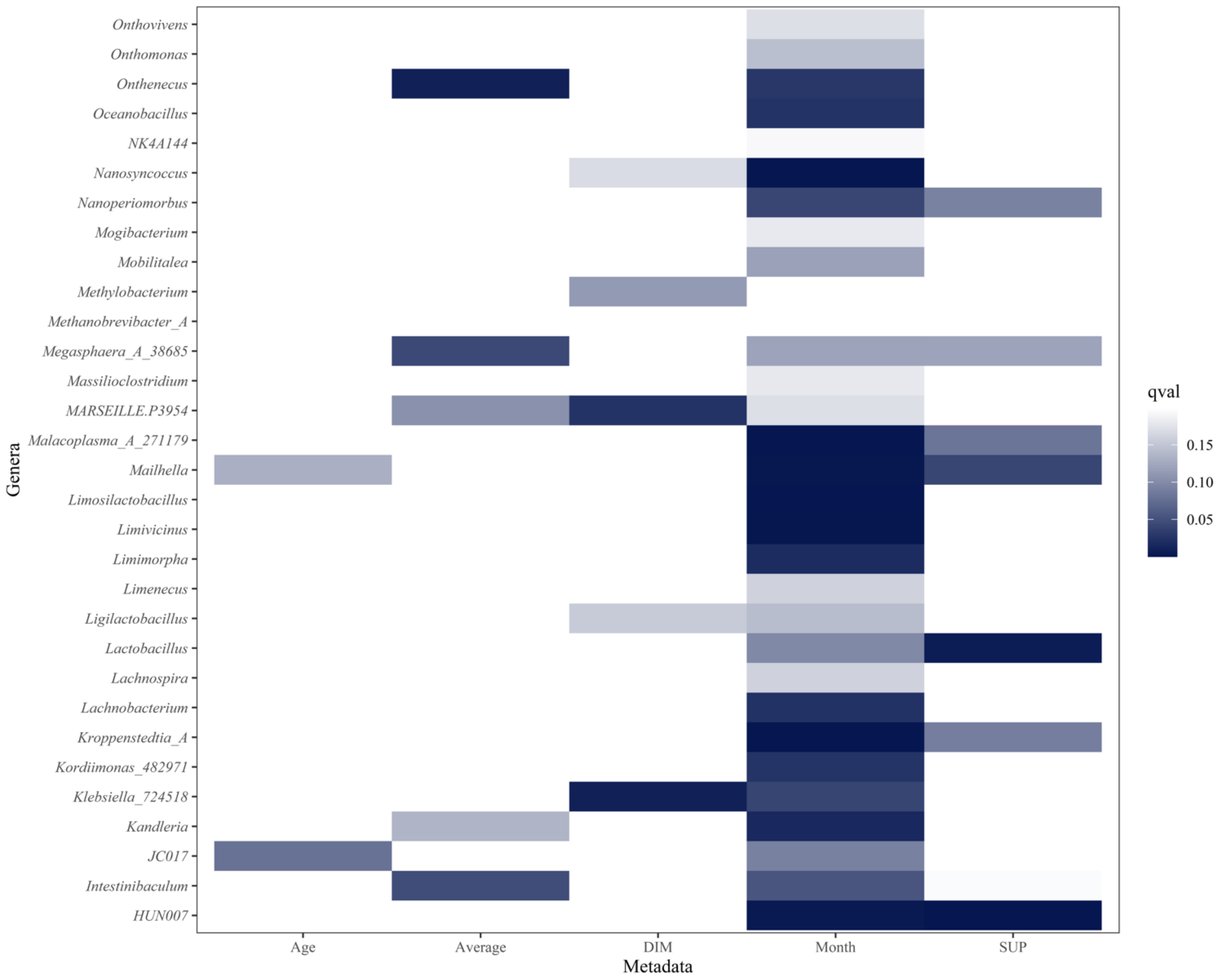

Figure S7: Heatmap of ruminal genera that are significantly associated with Age (years), Average milk (l/day), days in milk (DIM), calendar month (Month) and experimental group (SUP). Significant interactions ($P \leq 0.05$, FDR < 0.2) are colored in different shadings of red, with the most intense being the most significant. Genera are in reverse alphabetical order.

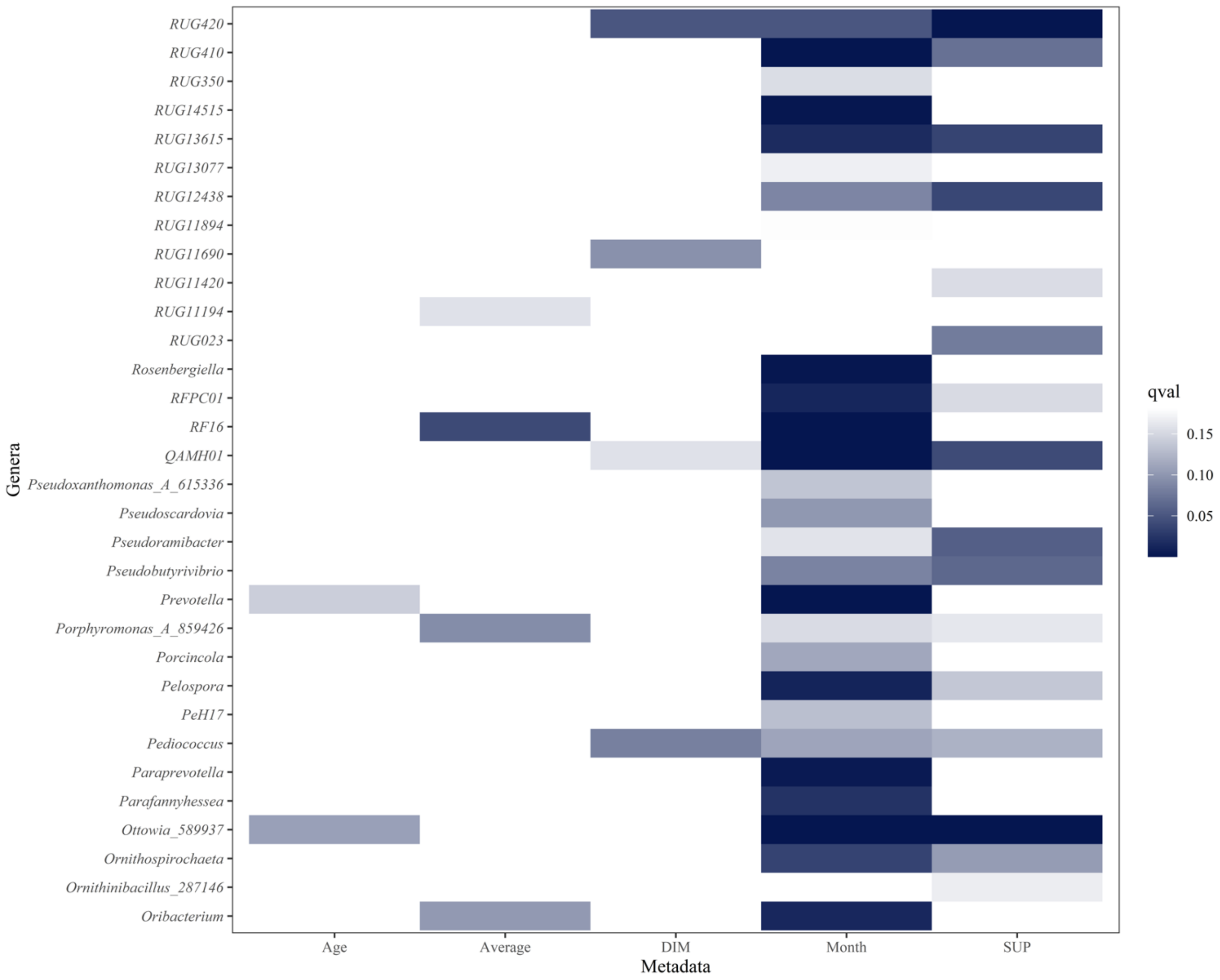

Figure S8: Heatmap of ruminal genera that are significantly associated with Age (years), Average milk (l/day), days in milk (DIM), calendar month (Month) and experimental group (SUP). Significant interactions ($P \leq 0.05$, FDR < 0.2) are colored in different shadings of red, with the most intense being the most significant. Genera are in reverse alphabetical order.

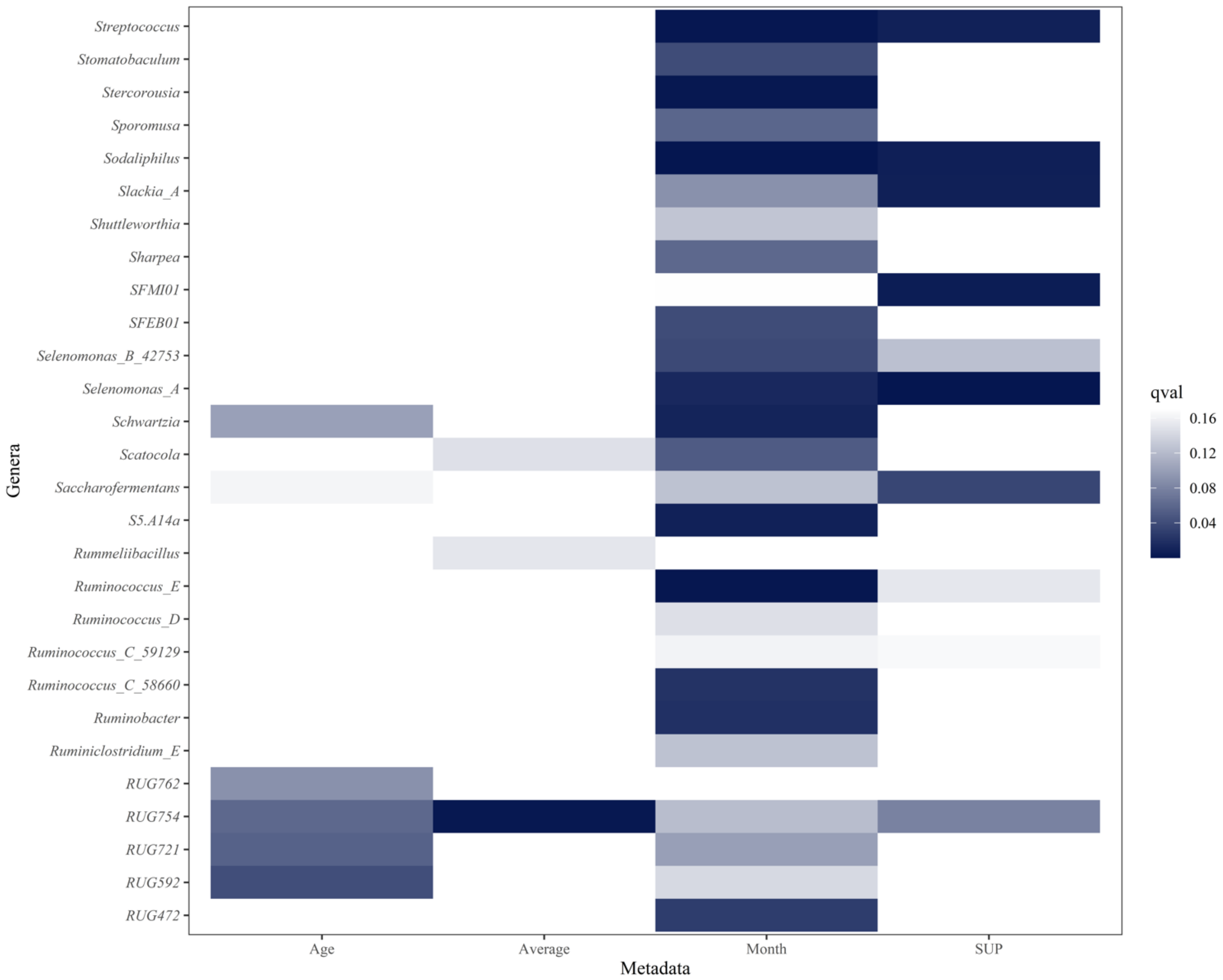

Figure S9: Heatmap of ruminal genera that are significantly associated with Age (years), Average milk (l/day), days in milk (DIM), calendar month (Month) and experimental group (SUP). Significant interactions ($P \leq 0.05$, FDR < 0.2) are colored in different shadings of red, with the most intense being the most significant. Genera are in reverse alphabetical order.

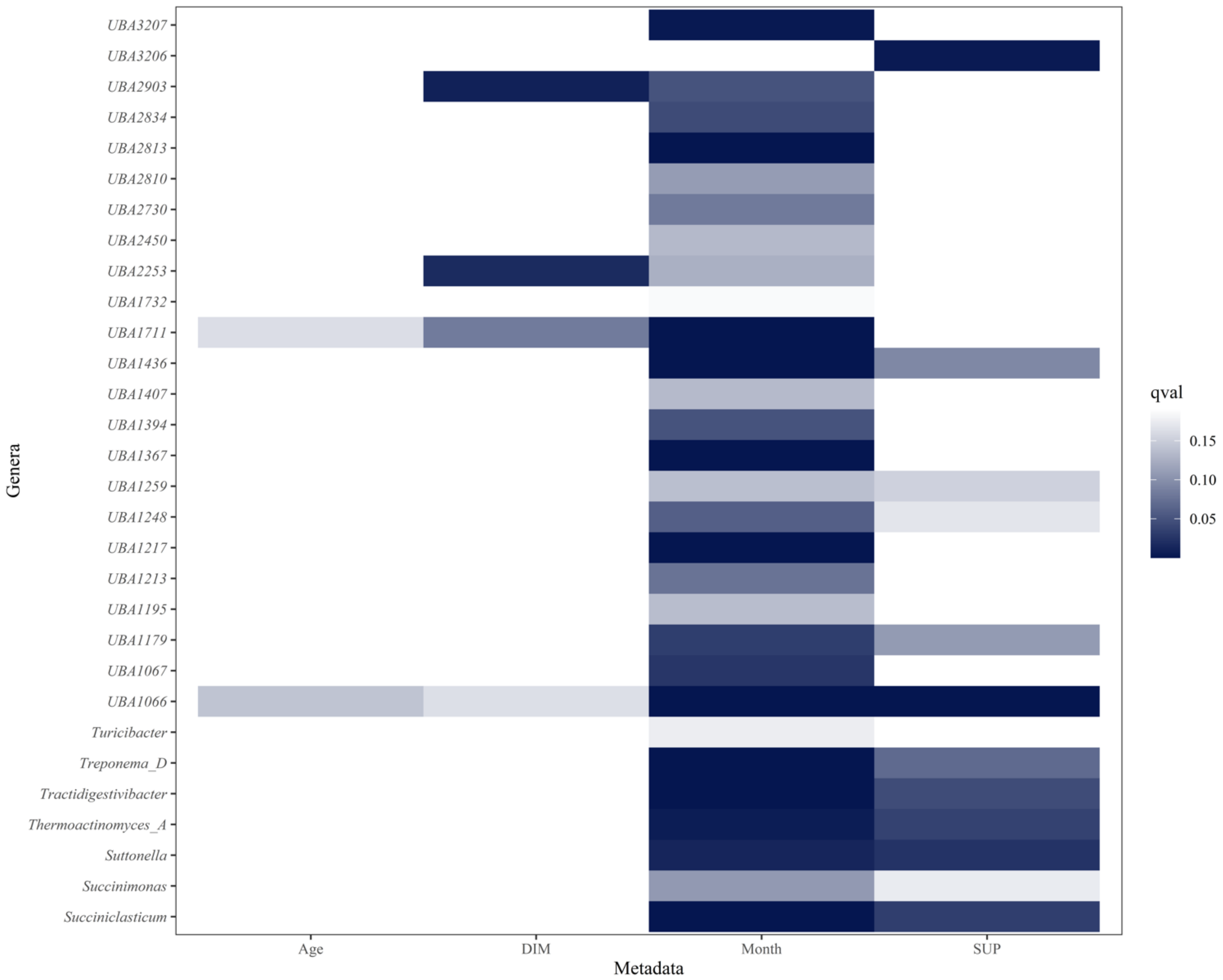

Figure S10: Heatmap of ruminal genera that are significantly associated with Age (years), Average milk (l/day), days in milk (DIM), calendar month (Month) and experimental group (SUP). Significant interactions ($P \leq 0.05$, FDR < 0.2) are colored in different shadings of red, with the most intense being the most significant. Genera are in reverse alphabetical order.

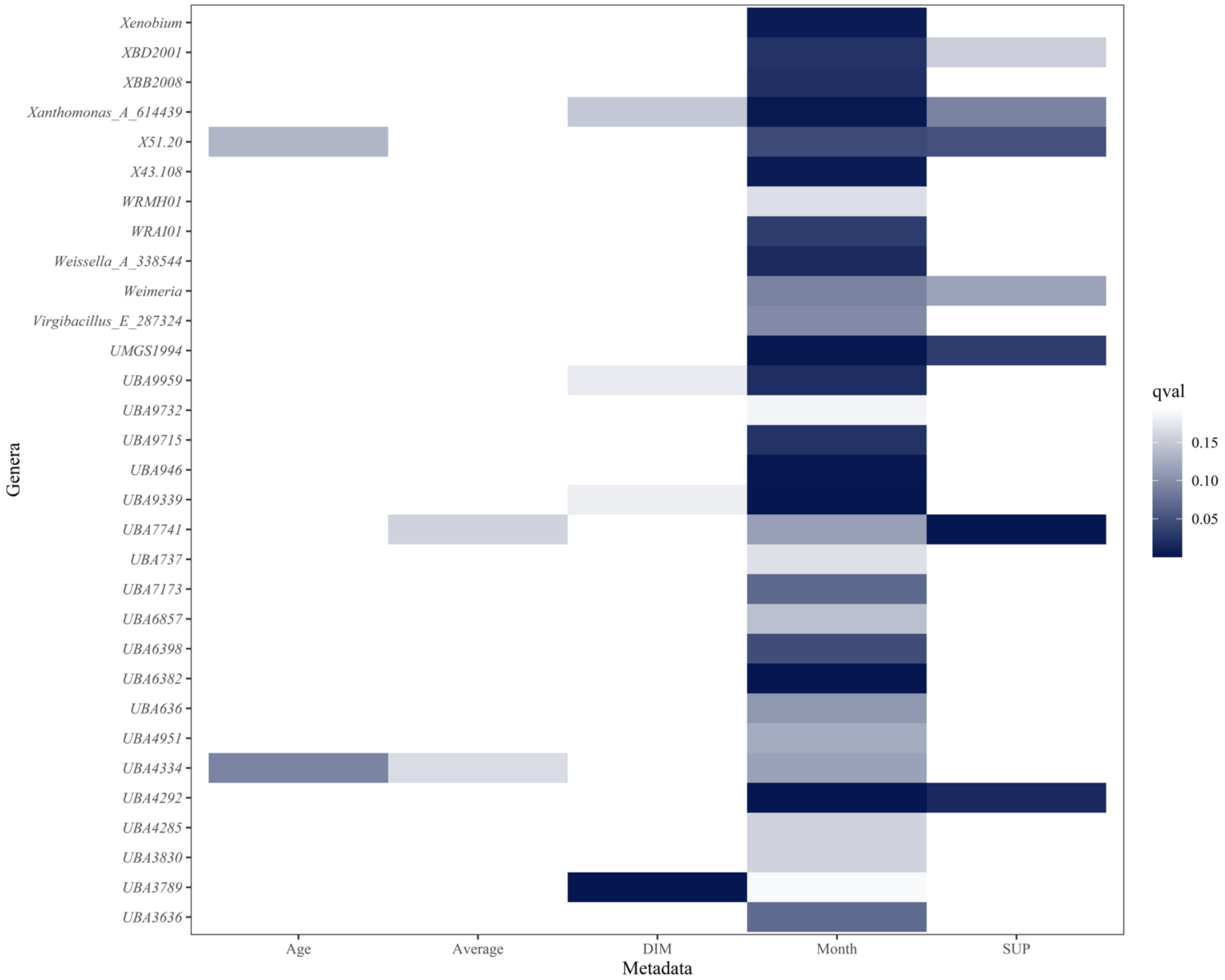

Figure S11: Heatmap of ruminal genera that are significantly associated with Age (years), Average milk (l/day), days in milk (DIM), calendar month (Month) and experimental group (SUP). Significant interactions ($P \leq 0.05$, FDR < 0.2) are colored in different shadings of red, with the most intense being the most significant. Genera are in reverse alphabetical order.
